# Functional Genomics and Comparative Genomics Analysis in Plants

**DOI:** 10.3390/cimb46120823

**Published:** 2024-12-05

**Authors:** Yueqi Lu, Quan Zou

**Affiliations:** 1 Yangtze Delta Region Institute (Quzhou), University of Electronic Science and Technology of China, Quzhou 324000, China; yueqi_lu@csj.uestc.edu.cn; 2 Institute of Fundamental and Frontier Sciences, University of Electronic Science and Technology of China, Chengdu 610054, China

The study of plant genomics has significantly deepened our understanding of plant evolution and adaptation from a microscopic perspective [1,2]. The abundance of plant genomic data has empowered researchers to uncover molecular-level insights into plants’ ecological adaptability, stress resilience, and pest and disease resistance, achieving meaningful advancements in plant breeding [3]. Following the analysis of gene functions, researchers can employ transgenic techniques or genome editing to facilitate precision breeding. Additionally, researchers can utilize comparative genomics to perform analyses of known genomic information, leveraging the highly homologous characteristics of related species to predict gene functions in non-model plants. In this Special Issue, scientists have contributed 19 papers that provide readers of *Current Issues in Molecular Biology* a novel perspective on research in plant functional genomics and comparative genomics.

Functional genomics research is crucial for the understanding of plant signal transduction and metabolic pathways, encompassing the analysis of transcriptomics, epigenomics, proteomics, interactomics, metabolomics, and phenomics. Hao et al. identified candidate genes for a high seed ratio (HSR) in *Camellia vietnamensis* through bulked segregant RNA (BSR) analysis and full-length transcriptome sequencing methods, revealing the potential roles of cytochrome P450, gibberellin phytohormone, and other pathways in the formation of HSR [4]. The key traits-related genes identified through functional genomics help achieve precision breeding to enhance crop yield and quality, as well as to improve specific nutritional components [5]. Rice genomics has revolutionized the field of rice biology and established rice as an excellent model organism for crop science research [6,7]. Xiang et al. conducted a genome-wide association study (GWAS) on root traits of 391 rice (*Oryz asativa* L.) accessions, identifying 10 new quantitative trait loci (QTLs) and highlighting the importance of epistasis in the inheritance of complex traits [8]. Wang et al. detected SNP loci associated with kernel number per row (KNR) in maize through bi-parental QTL mapping and GWAS, and identified three new candidate genes at this locus, providing a foundation for developing high-yielding hybrid varieties using heterosis patterns [9].

Moreover, researchers can investigate the functions of specific genes in plant growth, development, and environmental adaptation through gene knockout, overexpression, and transgenic technologies [10]. Li et al. have summarized the diverse applications of CRISPR/Cas9-mediated base editing technology in plant breeding, including precise single-nucleotide changes to produce desirable traits [11]. This technology holds great potential in increasing crop yield, improving quality, enhancing disease resistance, and promoting tolerance to herbicides.

Plant functional genomics is also extensively used in the identification of genes associated with disease resistance, pest resistance, and stress tolerance. Zhang et al. conducted an in-depth association analysis of the relationship between rice lesion mimic mutants and disease resistance, providing a theoretical basis and reference for mutants’ resistance to pathogens [12]. Li et al. identified nine significant SNPs associated with resistance to *Phytophthora sojae* in wild soybeans through a GWAS and predicted eight candidate genes [13]. Some studies improved the efficiency of functional gene enrichment by prioritizing functional genes for complex phenotypes employing genetics methods based on protein–protein interaction (PPI) network systems on available GWAS results [14]. Meanwhile, researchers also leverage plant functional genomics to study plant stress tolerance. Hasnaoui et al. conducted the de novo assembly and gene annotation of Greek mustard (*Hirschfeldia incana* L.), revealing key gene expression changes under lead stress [15]. Yang et al. performed a genome-wide identification and analysis of the calmodulin-like (CML) gene family in the salt-tolerant grass species Paspalum vaginatum, revealing the potential role of *Pv*CMLs in response to salt and cold stress [16].

The growing availability of plant genome sequences and transcriptome data has greatly facilitated interspecific comparative studies, including genomic analyses and gene co-expression network analyses [17]. Comparative genomics elucidates the structural and functional differences of genomes between different species, revealing their variations and adaptability throughout the evolutionary process. For example, researchers conducted comparative genomics analysis on *Panax* species and discovered that gene duplication plays a key role in the diversity of triterpenoid biosynthesis [18]. Through comparative analysis, scientists can simultaneously identify genes and regulatory elements related to important traits in plants, providing molecular-level guidance for crop improvement and breeding. Vu et al. sequenced and annotated the chloroplast genome of *Ficus simplicissima* Lour and conducted a comparative genomic analysis with five other species in the *Ficus* genus [19]. This study found the psbA-trnH gene region in the chloroplast genome as a potential candidate for chloroplast DNA barcoding, aiding in species identification within the *Ficus* genus. Yang et al. sequenced and comparatively analyzed the chloroplast genomes of 13 accessions of eggplant (*Solanum melongena*) and its wild relatives, revealing the precise phylogenetic relationships among them [20].

Overall, the papers in this Special Issue demonstrate the vital role of functional genomics and comparative genomics in plant science. Key genes associated with lead alleviation, cold and heat resistance, pest and disease resistance, developmental regulation, and high-yield traits have been identified across various species utilizing methods such as transcriptome analyses, chloroplast and mitochondrial genome sequencing, AFLP, GWAS, and gene family studies. These findings not only offer valuable genetic resources for subsequent functional studies and breeding efforts, but also provide updated insights into the molecular mechanisms and evolutionary background of the studied species. Given their potential implications for agricultural advancements and environmental sustainability, we hope such studies will receive more attention within the scientific community and among stakeholders.

Finally, we extend gratitude to the authors, reviewers, and staff at the *Current Issues in Molecular Biology* Editorial Office for their invaluable contributions.
